# In Situ VIS-NIR Spectroscopy for a Basic and Rapid Soil Investigation

**DOI:** 10.3390/s23125495

**Published:** 2023-06-11

**Authors:** Guillaume Debaene, Piotr Bartmiński, Marcin Siłuch

**Affiliations:** 1 Department of Soil Science Erosion and Land Protection, Institute of Soil Science and Plant Cultivation, State Research Institute, ul. Czartoryskich 8, 24-100 Puławy, Poland; 2 Department of Geology, Soil Science and Geoinformation, Institute of Earth and Environmental Sciences, Maria Curie-Skłodowska University, ul. Kraśnicka 2cd, 20-718 Lublin, Poland

**Keywords:** field measurements, near-infrared spectroscopy, PLS, SVM, soil properties, soil mapping

## Abstract

Visible and near-infrared (VIS-NIR) spectroscopy is extensively used in the field of soil science to predict several soil properties, mostly in laboratory conditions. When measured in situ, contact probes are used, and, very often, time-consuming methods are applied to generate better spectra. Unfortunately, spectra obtained by these methods differ greatly from spectra remotely acquired. This study tried to address this issue by measuring reflectance spectra directly with a fibre optic or a 4° lens on bare untouched soils. C, N content and soil texture (sand, silt, and clay) prediction models were established using partial least-square (PLS) and support vector machine (SVM) regression. With spectral pre-processing, some satisfactory models were obtained, i.e., for C content (R^2^ = 0.57; RMSE = 0.09%) and for N content (R^2^ = 0.53; RMSE = 0.02%). Some models were improved when using moisture and temperature as auxiliary data for the modelling. Maps of C, N and clay content generated with laboratory and predicted values were presented. Based on this study, VIS-NIR spectra acquired with bare fibre optic and/or a 4° lens could be used to build prediction models in order to obtain basic preliminary information on soil composition at the field scale. The predicting maps seem suitable for a fast but rough field screening.

## 1. Introduction

Visible and near-infrared spectroscopy (VIS-NIRS) is a rapid and non-destructive analytical technique that is extensively used in the agricultural sector [1]. The method is easy to use and allows the user to correlate reflected light with the soil’s chemical and physical properties [2]. The absorption in the NIR region is related to molecules that contain strong bonds between light atoms, such as C, H, O, and N. The method is both quantitative [3] and qualitative [4]. The predictions obtained by VIS-NIRS can be very accurate [5], and they thus allow for very precise mapping [6]. Moreover, they can often replace standard laboratory analyses [7]. The rise in the number of soil monitoring worldwide has triggered an important need for fast soil analyses, ideally in situ. Bulking soils to obtain a representative sample causes a loss of spatial information. There is, therefore, an urgent need to develop fast analytical methods that could also reduce analytical costs, thereby allowing an increase in the number of analysed samples.

VIS-NIRS is usually used under controlled laboratory conditions [8,9,10]. It is fast in comparison to classical analytical methods and is also environmentally friendly. However, building a laboratory-based spectral collection is still a laborious task due to the process of sampling preparation, i.e., drying, grinding, and sieving. The sample preparation stage is used to remove the influence of moisture content and soil structure. Sample preparation also makes the prediction models more accurate [11].

Not taking into account remote sensing, there are two possibilities to acquire field spectra: (i.) by using a mobile platform (i.e., Quraishi and Muazen [12], Knadel et al. [13]) or (ii.) by using handled or portable field instruments (i.e., Cozzolino et al. [14], Wenjun et al. [15]). However, there has been a lack of investigation of in situ applications of the method, which could allow the estimation of soil properties to be faster and to also detect spatial variability at a larger scale. Although mid-infrared (MIR) spectroscopy is showing some promise [16] for the accuracy of in situ calibration of soil organic carbon (SOC), the price of the instruments and the low availability of the field MIR spectrometer (generally handled FTNIR) is still a limitation to its implementation. Moreover, VIS-NIR spectra can be acquired airborne or even space-borne. VIS-NIR devices can be small and equipped with fibre optics, and can allow for the analysis of wet samples [17]. Furthermore, low-cost sensors are available. One of the preliminary VIS-NIR field studies was that of [18], which classified, with success, soil profiles using a VIS-NIR spectrometer with a specifically designed probe to scan the profile up to 1 m deep in a drilled hole. More recently [15], compared in situ (using a contact probe), laboratory-based models for several soil properties, including SOC, found similar results, especially when using the least-square support vector machine (LS-SVM) modelling. As underlined by Kühnel and Bogner [19], building a robust calibration under field conditions is a challenge. These authors have tried to incorporate field spectra into the calibration datasets (laboratory-based) in order to predict SOC in soil profiles. Using the SMOTE (synthetic minority oversampling technique) algorithm, they spiked the existing spectral library with new in situ spectra. This improved the prediction of SOC compared to models based on the spectral library alone. In another study, soil spectra were collected in situ in paddy soils [20]. The spectra have been used in combination with partial least-square regression (PLSR) in order to predict SOC content and other soil properties. SOC was successfully calibrated (R^2^ = 0.73; RMSE = 0.41). As mentioned earlier, Hutengs et al. [16] obtained moderately good results for in situ SOC prediction with MIR spectroscopy, but they failed with VIS-NIR (R^2^ = 0.39). However, the model root mean square error (RMSE) was rather small (0.23%), indicating an accuracy better than other studies with the same range of SOC values. All of these attempts to use the technology in situ have shown some promise, then, but they have almost always used a contact probe, which gives the mean spectra of only a very limited area (≈5 cm^2^), and which cannot be easily compared to remotely acquired spectra. The advantage of a contact probe is that it allows to not take into account ambient light. In the present study, we propose acquiring spectra in situ on bare soils using only a fibre optic or a 4° lens. The scanned areas thus represent a more representative sample of the soil surface. No special efforts were taken to improve the spectra. The main goal was to use the instruments for fast scanning.

Spectra collected in the field are different from laboratory ones [21], and also challenging to acquire due to the variations resulting from soil physical conditions, e.g., soil roughness and aggregates [22] and soil moisture content [23]. The main approach to deal with this problem is often to take the samples to the laboratory for drying and sieving in order to obtain controlled environmental conditions. Stevens et al. [24] have demonstrated that drying soil samples was enough to obtain better prediction accuracy for SOC. On the other hand, Wang et al. [25] have shown that model accuracies were better when wetting the samples, and they suggested that doing so could be a quick way to standardize the effect of moisture, since it is easier to wet soil than to dry it in the field. Several attempts to deal with the problem of moisture with in situ spectra were presented by Kühnel and Bogner [19]. The major drawback of these techniques is that they are rather difficult to implement due to the labour-intensive work and the statistical skills needed and/or the loss of some spectral information [26,27,28]. As has recently been underlined by Zhou et al. [29], collecting samples in the field for later laboratory work, eliminating moisture and particle size (soil texture) interferences takes too long for variable fertilisation. In situ operation is thus needed. If some equipment dedicated to working in the field (e.g., Spectral Evolution PSR-3500) is available, obtaining a quick result without supplementary laboratory work or intensive statistical investigation can at least be investigated. Perhaps, then, we should ask whether it is worth losing some of the model’s accuracy at the expense of simplifying the work?

The objective of this study was to test whether collecting VIS-NIR spectra in situ using a bare fibre optic and a 4° lens is suitable for determining various chemical and physical properties (C, N, and soil texture), which is particularly important not only in precision agriculture [30] but also in the context of climate change [31]. The scans of topsoil were collected on a grid sampling several fields of Eastern Poland.

## 2. Materials and Methods

### 2.1. Field Investigation

**Figure 1:**
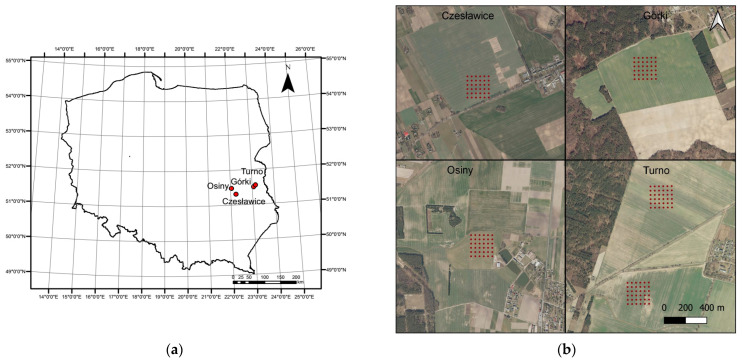
Study area in Poland (**a**) and sampling grid localisation at each site (**b**).

Four study sites located in the south-eastern part of Poland were selected for analysis: Czesławice, Górki, Osiny, and Turno (Figure 1). The study was conducted on regular soil grids (200 m × 200 m) with a resolution of 40 m (36 points). There were a total of five grids, one in Czesławice, Górki, and Osiny, and two in Turno (Figure 1). The field campaigns were held during the years 2018–2019 at different periods on bare soils (Table S1) for both the Górki and the Turno sites. Czesławice and Osiny were scanned a year earlier (10 October 2017 and 21 August 2017 to 13 October 2017, respectively). With the fibre optic and the lenses (4°), we obtained spectra from the soil surface only after removing pebbles, plants, etc. For the soil analyses (C, N, texture), the sampling took place at a depth of 10 cm, since it is difficult to obtain a sufficient amount of soil from just a few centimetres.

### 2.2. Sites Description

The Czesławice research area is located on an average slope, facing south. The soil cover consists of lessive soils made of total loess, partially eroded due to their location in relation to the topography. Osiny is located on a slight slope with southern exposure. The surface formations are Pleistocene glacial tills, sanded on the surface, with subsoil stagnant clays in the lower parts. In terms of typology, lessive soils with a clear profile grain size differentiation dominate. A smaller area is occupied by black soils with poor water conditions conditioned by heavy texture. The Górki site is located on a north-western slope. The surface is covered with brown and rusty soils, formed from sandy deposits lying on the glacial tills. In some places, Upper Cretaceous marls are revealed. The Turno 1 area is located on a slight slope with western exposure. The surface formations are glacial sands and gravels, and in the eastern part, glacial tills are present. Typologically, lessive soils dominate. Turno 2 soils are composed of sands and glacial gravels on the tills, and the soil cover consists of well-developed lessive soils, while the terrain has a slight westward exposition.

### 2.3. Physical and Chemical Analyses

The 36 samples from each sampling grid were analysed for C and N content, granulometry, temperature, and moisture at different periods of the year. Sometimes only 12 sampling points were taken for laboratory analyses due to the field management and the logistics involved. Carbon and nitrogen were analyzed using the LECO TruSpec analyser, taking into account the potential content of inorganic carbon in soils. The measurements were carried out in triplicate. Soil texture was analyzed by laser diffractometry on a Malvern Mastersizer 2000. At each scanning point of Górki and Turno 1 and 2, temperature and moisture were measured simultaneously during spectral acquisition by a time-domain reflectometry probe (Easy Test) combined with a temperature sensor.

### 2.4. Spectral Acquisition

Two spectrometers were used for spectroscopic measurements. The fields from Osiny and Czesławice were scanned using a spectroradiometer PSR-3500^®^ (Spectral Evolution Inc., Lawrence, MA, USA) in the 350–2500 nm range. For the scanning process to be faster and more similar to proximal or remote sensing acquisition, no contact probe was used. The spectra were acquired using a 4° lens from around 1 m high. No tripod was used either. The lack of precision was compensated by the faster acquisition time and the easier moves in the field. The instrument had a spectral resolution of 3 nm @ 700 nm, 10 nm @ 1500 nm, and 7 nm @ 2100 nm. It had one 512-element Si array and two 256 elements extended InGaAs arrays detectors with an internal scan time of 100 milliseconds. The reflectance data were interpolated to 1 nm by the software. The second instrument was an RS-3500, also from Spectral Evolution. The scanning with the RS-3500 took place at the sites of Górki and Turno (1 and 2) by using a bare fibre optic cable that was 1.5 m long. The instrument characteristics were similar to those described above. The light angle at the end of the fibre optic was 30° (information from Spectral Evolution). The end of the cable was held at around 30 cm above the soil. In both cases, the instruments were calibrated with a white Zenith Lite Target (95% diffuse reflectance) at each of the grid sampling series.

**Figure 2:**
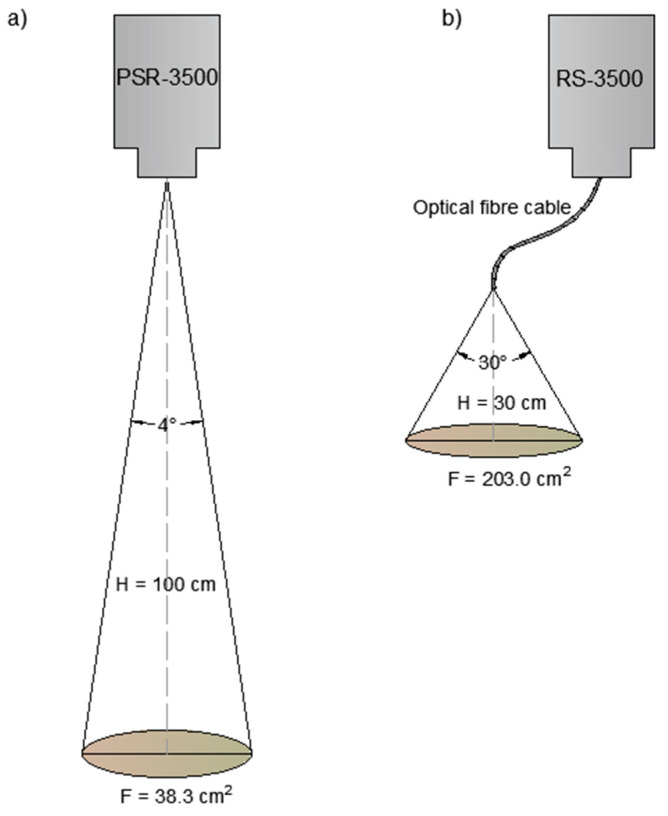
Areas scanned by the 4° lens with PSR-3500 Spectral Evolution spectrometer (**a**) and by the bare fibre optic with RS-3500 Spectral Evolution Spectrometer (**b**).

The scanning areas in the two cases are 38.3 and 203.0 cm^2^, respectively. This was believed to be more representative of the soil surface than the small area (5 cm^2^) covered by a contact probe (see Figure 2). Moreover, a contact probe will flatten the soil surface and alter its spectral properties (which does not happen with remote sensing).

No special care was given to prepare the soil before scanning given that we wanted it as similar as possible to air-borne/space-borne spectral acquisition. The only requisite was that the soils had to be without vegetation or visible pebbles. The spectra are an average of 30 consecutive spectra (software handled). The scans were measured in triplicate at each grid point, and the mean was then used as a representative spectrum. Spectra were truncated to 350–929 nm, 981–1829 nm, and 1941–2450 nm in order to (i.) eliminate noise at the end edge and (ii.) interferences at the contact between sensors or caused by water absorption [26]. Noises increase in a natural situation because of the ambient light, the distance of the fibre optic lens to the soil, the soil moisture, and the inherent soil surface heterogeneity.

### 2.5. Statistical Analyses, Modelling, and Mapping

Before modelling, all spectra were mean-centred and smoothed by moving the average (MA) with a segment size of 7, and they were pre-processed with standard normal variate (SNV) and multiplicative scatter correction (MSC) to reduce the variability between the samples due to light scattering and baseline shifts, which are significant in field samples and with Savitzky-Golay (SG) first derivate (see Rinnan et al. [32] for more detail on these pre-processing methods). SG derivatives smooth the spectra and can enhance signal properties or suppress unwanted features [33]. A principal component analysis was applied to the pre-processed (MA) spectra to investigate spectral variability and identify an eventual relation between the samples at each site.

All the calibrations were built using partial least square (PLS) regression, and the support vector machine (SVM) regression using 3-fold cross-validation (CV) with random segments. CV was used due to the low number of samples available at each site. Only the best combination of pre-processing and calibration will be presented here. All pre-processing and models were developed with Unscrambler v.10.3 (Camo Analytics, Oslo, Norway). PLS regression (see [34]) is one of the most popular methods used with VIS-NIR spectroscopy, and it aims to determine the best correlation between the soil properties and the spectral data. This is a reduction dimension technique that seeks a set of latent variables by maximising the covariance between the spectra (X) and the soil properties (Y). The goal of PLS regression is to predict Y from X. The number of factors used was 7 due to the size of the dataset. SVM is often used for classification, but it can also be used for regression [35]. It is a nonlinear calibration method based on statistical learning with no assumption for normality.

The models were evaluated by using R^2^ value (references vs. predicted values), root mean square error of cross-validation (RMSECV), and the ratio of standard deviation (RPD). Maps were created with ArcGIS 9.3 (ESRI, Redlands, CA, USA) using natural neighbour (NN) interpolation.

## 3. Results and Discussion

### 3.1. Physical and Chemical Properties of Soils

The mean moisture content and mean soil temperatures available at each site during the scanning session are presented in Table S1. The range of moisture and temperature during spectral acquisition was 6.1% to 25.6% and 6.7 °C to 31.7 °C, respectively. There was a negative correlation between the two values (R^2^ = −0.49). Summary statistics of the analysed soil properties are presented in Table 1. The soil properties are typical for soils of the region [6,36,37]. The average C content of the investigated fields is comprised between 0.95% ± 0.11% and 1.18% ± 0.13%. Nitrogen content is between 0.05% ± 0.02% and 0.08% ± 0.02%. The different sites have very similar C and N contents. All sites are composed of the loamy sands that are very common in the region [8], except for Czesławice, which is a silt loam (according to the USDA classification). Hence, the results can be compared, and the impact of soil texture on soil reflectance can be minimised. We hypothesise, therefore, that the main factors affecting the spectra, except for ambient light and the soil surface, were the SOC content and the soil moisture.

### 3.2. Spectra Description

**Figure 3:**
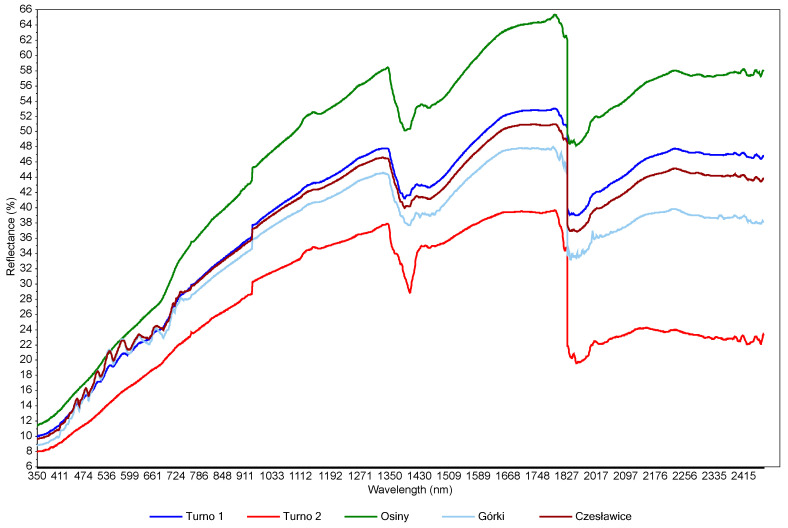
Mean spectra for the four investigated sites.

The mean spectra for each site are presented in Figure 3. All VIS-NIR spectra were similar in both shape and peak localisation. Despite the difference in texture (Table 1), Czesławice mean spectra is also similar to the other site spectra. This is probably due to the soil moisture hiding features related to clay and silt. There was a reflectance increase with increasing wavelength (from 350 to 1350 nm). In the visible range (350–780 nm), the reflectance is mostly associated with iron from minerals and soil chromophores from organic matter [3]. The shape of the spectra in that visible region did not change significantly, and it is probably the result of different water content. The water absorption from –OH bonds is visible at 1400 nm. The 1900 nm peak was deleted during the pre-processing stage due to the very strong noise related to soil moisture (strong water absorption in that region and noise from atmospheric water vapor [38]). Some small peaks are visible at around 2200 nm and 2300 nm and are related to clay minerals [39]. The main differences were observed in the baselines and peak intensity due to the moisture variations, and probably also due to the C content and the texture differences between the sites. The spectra are typical for wet mineral soils [40]. A more detailed study of the spectra could be undertaken on an individual spectrum rather than a mean spectra as more distinct absorption features are observable, but this is not the focus of this paper. It was also observed that measurements (spectra) located where the moisture content was more heterogeneously distributed in the field presented more variations and more noises. This underlined, one more time, the disturbing effect of soil moisture on the spectra. This is caused by light absorption by the water, which can cause spectral variations as described in [41].

**Figure 4:**
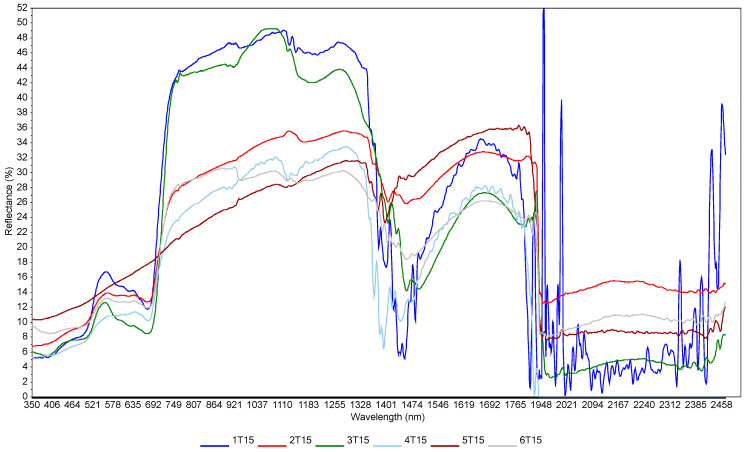
Spectral variation related to moisture content at Turno 2 site. These are spectra taken at the same point at different scanning sessions. The moisture contents were: 1T15—25%; 2T15—19%; 3T15—20%; 4T15—15%; 5T15—7%; 6T15—9%. Numbers from 1 to 6 indicate the sampling dates; T indicates Turno 2 study site; and 15 indicates sample point no. 15 from the 36 grid points.

**Figure 5:**
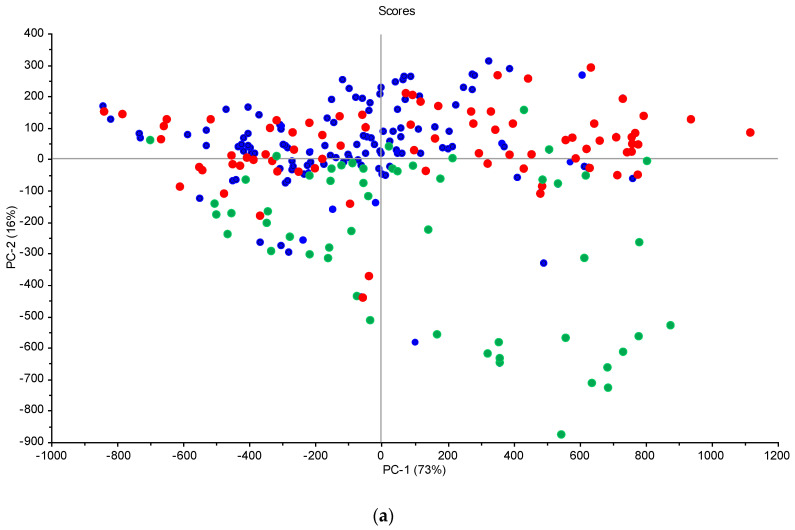

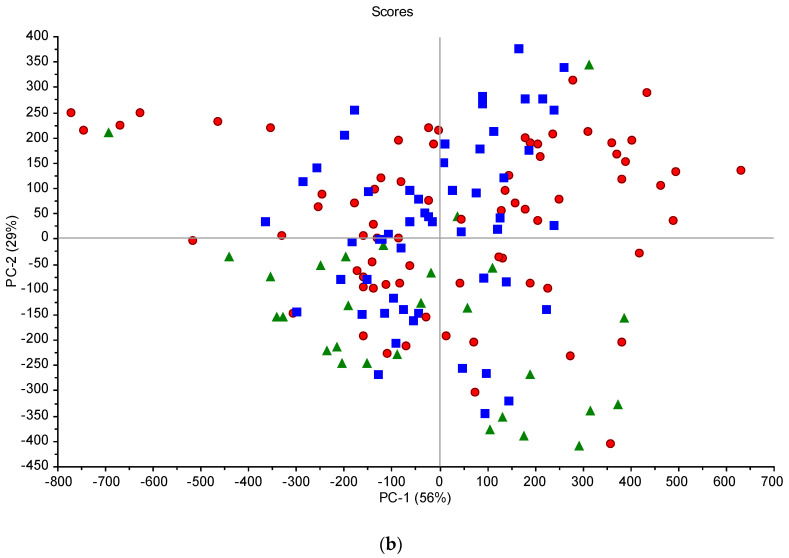
Score plots from the PCA analysis of the spectral data for the Górki (**a**) and Turno (**b**) sites—green circles/triangles, low moisture; red circles, average moisture; blue circles/squares, high moisture.

The effect of moisture is visible (Figure 4), which represents spectra acquired at one point (sample 15 from the grid in Turno 2) but at different scanning sessions (six sessions). Figure 3 only aims to showing the effect of soil moisture on the spectra, since no models could be developed by using combined scanning sessions. There are great variations in the localization and the intensities of the peaks (e.g., in the visible range). This is the result of soil moisture, sun illumination intensity, surface heterogeneity and the possible presence of small plant cover or pebbles. This underlines the limitations of the method when scanning samples under different meterological and soil conditions. There is a clear trend of increasing reflectance when the moisture content is increasing in the 700–1400 nm range. Typically, sample outliers, such as, for example, 1T15, were removed from the modelling. This trend was present at each site, even if, sometimes, other factors such as SOC content or soil texture rendered the trend not perfect. This has been described at length in many papers (e.g., Knadel et al. [42], Biney et al. [40]). In the principal component analyses (PCA) (Figure 5), moister (blue) samples are mostly in the upper part of the graph, and samples with low water content (green) are in the lower quadrants of the plots. The two score plots (Figure 5) are similar in shape, but the clustering according to moisture content seems to be site-specific (there is a better grouping in the Górki site despite the very similar range of moisture). This is due to the drying process, which tends to move the sample along the axis of the score plot, as described in Debaene et al. [43]. The same was observed for the two sites (Figure 5a,b) but also at each scanning session separately. Samples are seen moving to the lower quadrants of the score plots when the moisture content is lower. This was also observed in Roudier et al. [44] on soil samples with a wide range of SOC and clay content from soil cores in New Zealand. Unfortunately, no moisture and temperature data were available for the two other sites (Czesławice and Osiny).

### 3.3. Model Evaluation and Mapping

The advantage of PLS over SVM regression is that it allows for a better interpretation of the results. There were very few differences between PLS and SVM regression in our study, with SVM models somewhat less robust, and, therefore, only PLS models are presented here (see Table 2). This was reported many times in the literature: Igne et al. [45], for example, investigated VIS-NIR and mid-infrared spectra of dry and wet field samples (ultisols) from Maryland, USA with several instruments in order to predict total C and N, sand, silt, and clay content, and Shi et al. [46] did not observe any significant differences between the methods for the prediction of total nitrogen of 100 samples from different soil types in China. This is the case for small datasets, such as the ones in the present study, but probably not for larger ones with a lot of organic matter and particle size variation [47]. The best SOC model was obtained in Osiny (R^2^ = 0.53; RMSE = 0.16; RPD = 1.4). An RPD value of 1.4 is considered a satisfactory model according to the classification by Chang et al. [48].

Generally, there was an underestimation of SOC values in the higher range in the silt loam field, and there was over-prediction in loamy sands. This was also reported by Stenberg [49]. Silt and clay were predicted with the best results in Czesławice, probably due to the fact that the soils there are composed mostly of silts with very little sand content.

All models failed to predict sand, which is often difficult to model since quartz is nearly transparent to the NIR radiation and, therefore, presents no significant features in the VIS-NIR range investigated here [50]. Though the effect of temperature is documented on the VIS-NIR spectra [28,51], there are very few studies that take into account the temperature when modelling. Knadel et al. [52] reported that fusing temperature data with spectral data improved the prediction accuracy for SOC when scanning a field on-the-go with a mobile platform on a farm with high SOC variability in Denmark. In another paper, Knadel et al. [13] increased the accuracy when predicting clay content in a field of the same farm in Denmark by fusing spectral data and temperature. In both cases, the range of temperature variation was about 5°, which is the same as that in the present study. Moreover, they found that fusing the electrical conductivity with temperature in a similar fashion further improved the accuracy. Unfortunately, we do not have EC data. One interesting observation was that the temperature was negatively correlated with SOC (R^2^ = −0.56), as can be found here (R^2^ = −0.49). Therefore, temperature is also a factor affecting field VIS-NIR acquired in situ, and it should be taken into account. When possible, it is thus interesting to measure the soil temperature at the sampling point in order to incorporate data in the modelling. This is, of course, time consuming, and it is easier to implement when using an on-the-go mobile platform.

Kuang and Mouazen [53] also demonstrated that soil moisture, sand, silt, and clay have a negative effect on the accuracy of SOC and N content in situ (on-the-go). They observed that on the wet samples (field samples), when the clay content increased, the moisture increased and the RPD decreased. This was explained by the fact that clay can hold a large amount of water, which can deteriorate the spectra. Therefore, the accuracy of C and N content was lower in that case. Stenberg [49], on the contrary, found that the accuracy was better with the clayed samples. We did not observe many differences between the silt loam and the loamy sand in this study.

The soil properties most strongly correlated with the VIS-NIR spectra were C and N content. This is often the case in the literature [54], and it is related to the fact that light is absorbed by, to take one example, C–H or N–H bonds that are present in organic compounds. The texture of the soil was less successful for prediction purposes, although the texture is often well predicted, such as in [55]. In most papers, sand models will be less robust than clay models [56], as it is the case here. However, sand is sometimes difficult to predict, as far as we understand it, due to the high Si content. No other papers have been found in the literature scanning in situ with bare fibre or a 4° lens.

A few models were improved by incorporating auxiliary data (temperature and soil moisture). Improvement of C and N content N models could be related to water retention by organic matter. On the other hand, though, soil temperature as auxiliary data only improved the prediction of clay and silt content.

**Figure 6:**
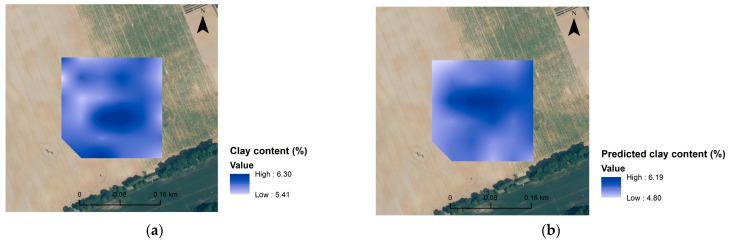

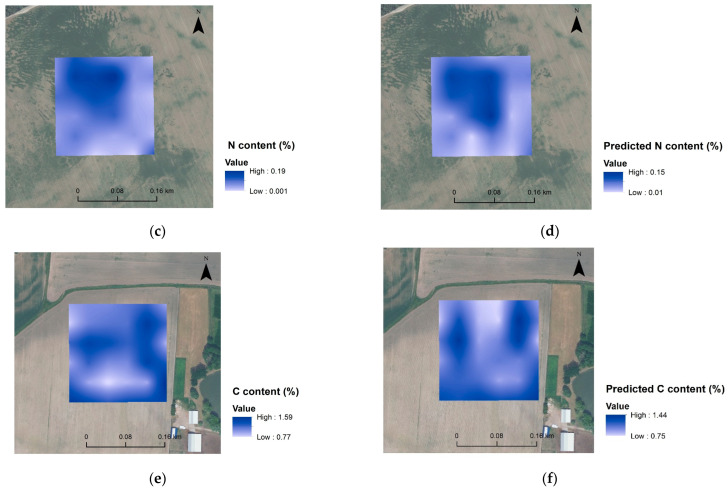
Spatial distribution maps of analysed clay content (**a**) and predicted clay content (**b**) for Czesławice; analysed N content (**c**) and predicted N content (**d**) for Górki, and analysed C content (**e**) and predicted C content (**f**) for Osiny.

**Figure 7:**
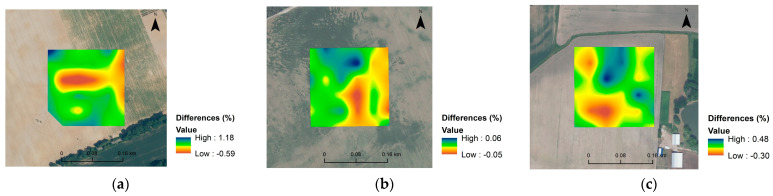
Spatial distribution maps of differences between predicted and measured clay content (**a**) for Czesławice, N content for Górki (**b**), and C content (**c**) for Osiny.

It has been demonstrated elsewhere [8] that only satisfactory VIS-NIR models for SOC and Clay content could produce very precise maps at the field scale. Consequently, maps of C, N and clay contents were generated here for some of the more robust models. The maps (Figure 6) are overall similar, as can be seen in Figure 7, and the differences in spatial distribution between predicted and measured values are presented, roughly providing an idea of the variation of C, N and clay content in the investigated area. These maps are probably not precise enough to be used for precision agriculture, but they do provide a fast overview of the field characteristics.

Better results, we can note, were obtained at both the Czesławice and the Osiny sites (see Table 2). These two fields were scanned using the 4° lens instead of the bare fibre optic. The 4° lens is directly attached to the instrument, which is held on the shoulder at a constant height (100 cm). On the other hand, the fibre optic cable was held around 30 cm from the soil surface, which induced some possible height discrepancies between the measurements. Moreover, the surface scanned with the lens is five-fold the surface scanned with the fibre optic (see Figure 2), and it is, therefore, more representative.

The method investigated here (i.e., the use of fibre and lenses) is simple and easy to implement in the field, unlike other solutions proposed in the literature, such as using a kind of black box/chamber to counteract the noises coming from ambient light [57,58] or using the mobile platform that required a tractor (e.g., Knadel et al. [13]).

## 4. Conclusions

In this study, we have investigated the possibility of using a VIS-NIR spectrometer to measure soil reflectance in situ in fields in Eastern Poland. Bare soils were scanned, and no attempts were made to correct for either soil moisture or ambient light. The spectra were used to predict C, N and soil texture. Carbon content was the best-predicted property, followed by N content. Relatively satisfactory prediction models were obtained at the Czesławice site for C, N and clay content, and at the Osiny site for C and N content. Silt and sand models failed at all sites, but at Czesławice with R^2^ = 0.61 and RMSEcv = 1.11. The main findings were that: (1) there are no significant differences between PLS and SVM regression methods; (2) auxiliary data, such as soil temperature and soil moisture, can sometimes improve the prediction models; (3) pre-processing the spectra is necessary to improve the accuracy, even though only satisfactory models were obtained; and (4) crude maps of soil variability could be produced with the predicted values.

Finally, the models are at best satisfactory. This is related to the effect of moisture and uncontrolled ambient light. Some of the limitations of the method are that VIS-NIR acquisition in the field with fibre optic or the 4° lens cannot be performed with either changing weather or when the soil moisture is too high. The best option, then, is to scan when the soils are dry before the formation of crusts or surface aggregates. In order to prevent the effect of ambient light, a portable chamber could be used. There should also always be an attempt to organize field scanning when the cloud coverage is both minimal and stable. Finally, the measurement height should be as precise as possible. More investigations are still needed on this subject in the future.

## Figures and Tables

**Table 1:** Summary statistics of the soil properties for the investigated sites.

Localisation	Czesławice	Górki	Osiny	Turno 1	Turno 2
**Total C content (%)**					
Min	0.72	0.54	0.75	0.75	0.85
Max	1.25	3.44	1.61	1.36	1.50
Mean	0.95	1.16	1.12	1.02	1.18
SD	0.11	0.54	0.23	0.12	0.13
Q1	0.88	0.79	0.93	0.94	1.10
Q3	1.03	1.30	1.29	1.09	1.25
**Total N content (%)**					
Min	0.05	0.00	0.03	0.02	0.04
Max	0.24	0.19	0.13	0.10	0.11
Mean	0.08	0.06	0.07	0.05	0.07
SD	0.02	0.04	0.02	0.02	0.01
Q1	0.06	0.03	0.06	0.04	0.06
Q3	0.08	0.09	0.09	0.06	0.08
**Sand content (%)**					
Min	16.7	50.1	52.0	70.9	67.3
Max	26.8	88.0	87.4	83.5	82.2
Mean	21.4	74.2	72.6	77.4	73.8
SD	1.7	9.2	9.1	2.8	2.2
Q1	20.3	68.3	66.3	75.5	72.6
Q3	22.4	81.8	79.6	79.1	74.9
**Silt content (%)**					
Min	68.4	11.2	11.6	15.4	16.8
Max	77.7	45.0	43.8	27.3	30.2
Mean	73.2	23.9	25.1	21.2	24.4
SD	1.4	8.4	8.3	2.6	2.0
Q1	72.4	16.7	18.8	19.5	23.4
Q3	74.1	29.6	31.1	22.8	25.5
**Clay content (%)**					
Min	4.7	0.8	0.8	1.1	1.0
Max	6.3	4.9	5.6	2.1	2.4
Mean	5.4	1.9	2.4	1.5	1.7
SD	0.4	0.8	1.1	0.2	0.2
Q1	5.1	1.3	1.5	1.3	1.6
Q3	5.7	2.2	2.9	1.6	1.8
Texture USDA	Silt Loam	Loamy Sand	Loamy Sand	Loamy Sand	Loamy Sand

SD—Standard deviation; Q1—25th percentile; Q3—75th percentile.

**Table 2:** Results from calibration models.

Study Site	Soil Properties	R^2^	RMSECV	RPD	NF	Pre-Processing	Auxiliary Data
Czesławice	C	0.57	0.09	1.22	3	SNV	
	N	0.53	0.05	1.33	4	SNV	
	Sand	0.33	2.28	0.74	4	MA	
	Silt	0.61	1.11	1.27	5	SNV	
	Clay	0.59	0.32	1.25	7	MSC	
Górki	C	0.47	0.6	0.9	2	SNV	b
	N	0.39	0.05	0.81	5	SG	-
	Sand	0.42	11.08	0.83	5	SNV	-
	Silt	0.48	9.13	0.92	5	MA	a
	Clay	0.51	0.65	1.19	3	SNV	a
Osiny	C	0.52	0.16	1.4	4	MA	-
	N	0.55	0.01	1.4	4	MSC	b
	Sand	0.21	15.2	0.6	5	MA	-
	Silt	0.37	10.37	0.8	5	MA	-
	Clay	0.34	1.37	0.8	5	MA	a
Turno 1	C	0.41	0.14	0.84	6	SNV	-
	N	0.18	0.04	0.51	1	MA	-
	Sand	0.31	3.94	0.71	5	MA	-
	Silt	0.35	3.56	0.73	5	SNV	a
	Clay	0.28	0.29	0.68	5	SNV	a
Turno 2	C	0.43	0.13	1.0	4	SNV	-
	N	0.23	0.02	0.52	6	MA	b
	Sand	0.29	4.07	0.54	7	MA	-
	Silt	0.19	4.08	0.49	7	MA	-
	Clay	0.37	0.25	0.81	4	SG	a

NF—Number of factors for the PLS regression; MA—Moving average; SNV—Standard normal variate; MSC—Multiplicative scatter correction; MSC—Savitzky-Golay first derivative; a—Improved with temperature data; b—Improved with soil moisture data.

## Data Availability

The raw data presented in this study are available on request from the corresponding author. The data are not publicly available due to intellectual property.

## Supplementary Materials

The following supporting information can be downloaded at: https://www.mdpi.com/article/10.3390/s23125495/s1, Table S1: Scanning and sampling sessions with moisture and temperature mean for Górki and Turno sites.
