# Supplementary material for: In Situ VIS-NIR Spectroscopy for a Basic and Rapid Soil Investigation
Source: Sensors (Basel). 2023 Jun 11;23(12):5495. doi: 10.3390/s23125495 (PMC10303191; doi:10.3390/s23125495)
Supplement: Supplementary file 1 [file sensors-23-05495-s001.zip › sensors-2431667-supplementary.pdf]

Table S1. Scanning and sampling sessions with moisture and temperature mean for Górki and Turno sites.

Site	No of samples	Date	Moisture (%)		Soil temperature (°C)	
			**Mean**	**SD[1]**	**Mean**	**SD[1]**
Górki	36	04.04.2018	20.7	6.6	12.9	2.0
	12	04.05.2018	6.1	2.6	26.8	1.3
	12	22.05.2018	13.8	2.8	23.9	1.2
	36	30.10.2018	19.2	5.9	13.9	0.9
	12	08.11.2018	19.9	4.8	10.6	0.7
	36	19.03.2019	21.3	5.9	6.7	0.8
	36	02.04.2019	14.4	6.9	9.3	2.3
	36	17.04.2019	11.5	4.1	21.8	1.0
	36	31.05.2019	12.5	4.6	31.7	1.2
Turno 1	36	29.08.2018	9.2	1.7	19.4	1.2
Turno 2	36	04.04.2018	25.6	3.8	14.2	0.4
	36	17.04.2018	19.1	3.0	14.9	0.2
	12	04.05.2018	10.7	2.2	31.4	1.4
	36	30.10.2018	19.0	3.3	11.5	0.8
	36	19.03.2019	15.7	2.7	7.9	0.7
	36	10.09.2019	6.9	1.3	23.7	1.3
	36	17.10.2019	9.0	2.4	21.8	1.1

[1] Standard deviation.
